# Physiological aspects of nitro drug resistance in *Giardia lamblia*

**DOI:** 10.1016/j.ijpddr.2018.04.008

**Published:** 2018-04-28

**Authors:** Joachim Müller, Andrew Hemphill, Norbert Müller

**Affiliations:** Institute of Parasitology, Vetsuisse Faculty, University of Bern, Länggass-Strasse 122, CH-3012 Bern, Switzerland

**Keywords:** Adaptation, Anaerobiosis, Diplomonads, Oxidoreduction, ALB, albendazole, MTZ, metronidazole, NTZ, nitazoxanide

## Abstract

For over 50 years, metronidazole and other nitro compounds such as nitazoxanide have been used as a therapy of choice against giardiasis and more and more frequently, resistance formation has been observed. Model systems allowing studies on biochemical aspects of resistance formation to nitro drugs are, however, scarce since resistant strains are often unstable in culture. In order to fill this gap, we have generated a stable metronidazole- and nitazoxanide-resistant *Giardia lamblia* WBC6 clone, the strain C4.

Previous studies on strain C4 and the corresponding wild-type strain WBC6 revealed marked differences in the transcriptomes of both strains. Here, we present a physiological comparison between trophozoites of both strains with respect to their ultrastructure, whole cell activities such as oxygen consumption and resazurin reduction assays, key enzyme activities, and several metabolic key parameters such as NAD(P)^+^/NAD(P)H and ADP/ATP ratios and FAD contents. We show that nitro compound-resistant C4 trophozoites exhibit lower nitroreductase activities, lower oxygen consumption and resazurin reduction rates, lower ornithine-carbamyl-transferase activity, reduced FAD and NADP(H) pool sizes and higher ADP/ATP ratios than wildtype trophozoites. The present results suggest that resistance formation against nitro compounds is correlated with metabolic adaptations resulting in a reduction of the activities of FAD-dependent oxidoreductases.

## Highlights

• Physiological comparison between susceptible and nitro drug resistant *Giardia lamblia* trophozoites.
• Resistant trophozoites exhibit lower nitroreductase activities, lower oxygen consumption and lower resazurin reduction rates.
• Resistant trophozoites exhibit lower FAD and NADP(H) contents and higher ADP/ATP ratios.
• Resistance formation against nitro compounds correlated with metabolic adaptations avoiding nitrosative and oxidative stress.

## Graphical abstract

**Figure:**
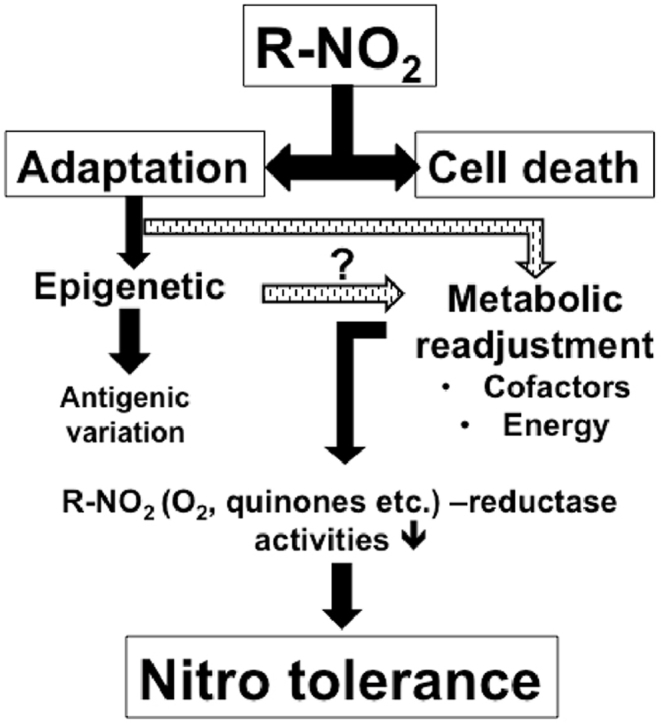

## 1 Introduction

*Giardia lamblia* (syn. *G. duodenalis*; *G. intestinalis*), a flagellated, amitochondrial, binucleated protozoan, is the most common causative agent of persistent diarrhea worldwide (Ankarklev et al., 2010; Carranza and Lujan, 2010; Einarsson et al., 2016; Müller and Müller, 2016). Giardiasis is commonly treated with metronidazole (MET), other 5-nitroimidazole compounds (Minenoa and Avery, 2003), nitazoxanide (NTZ) or albendazole (ALB) as an alternative in the case of resistance to nitro drugs (Nash, 2001; Solaymani-Mohammadi et al., 2010; Hemphill et al., 2013). Moreover, *G. lamblia* is susceptible to a variety of antibiotics because of its prokaryote-like transcription and translation machineries (Müller and Hemphill, 2013). According to a commonly accepted model, nitro compounds are activated by reduction yielding toxic intermediates, the electrons being provided by pyruvate oxidoreductase (POR). The reduced nitro compound then binds covalently to DNA and results in DNA breakage and cell death (Brown et al., 1998). Resistance formation to nitro compounds is, however, eagerly detected both *in vitro* and *in vitro.* Studies with metronidazole-resistant strains have revealed, however, that resistance is not always correlated with reduced POR activity thus mechanisms of action independent of POR activity may exist (Upcroft et al., 1990; Upcroft and Upcroft, 1993; Ansell et al., 2015; Leitsch, 2015).

In accordance to the prevailing model for the mode of action of nitro drugs, one would hypothesize that resistant trophozoites have decreased activities of nitroreductases, and that this decrease is due to lower expression levels of the corresponding genes. To verify this hypothesis, freshly obtained, resistant patient isolates would be optimal, but they are difficult to maintain in axenic culture. Therefore, most of the studies compare resistant “model” strains generated *in vitro* with isogenic wildtype strains (Upcroft, 1998). These studies have revealed genome rearrangements (Upcroft et al., 1990, 1992) and profound transcriptional changes evidenced by differential analyses using microarrays followed by quantitative RT-PCR on selected transcripts (Müller et al., 2008) and strand-specific RNA sequencing (Ansell et al., 2017). In both studies, expression profiles of genes coding for variant surface proteins and for genes involved in oxido-reductions – amongst others - are altered the latter allegedly confirming this hypothesis.

These studies on transcriptional changes do not reveal, however, the alterations that occur with respect to the cellular physiology of the resistant lines. Questions such as whether these lines have reduced reductase activities only with nitro drugs or also with other compounds as electron acceptors, and whether they have different pool sizes or ratios of electron and energy providing cofactors, need to be addressed. In this study, we document the physiological changes during resistance formation to nitro drugs in *G. lamblia*, comparing a nitro drug-resistant strain, namely the previously introduced strain C4 (Müller et al., 2007, 2008) and its corresponding wild-type (WBC6) with respect to their ultrastructure, whole cell activities such as oxygen consumption and resazurin reduction assays, functional assays, and pool sizes and ratios of cofactors involved in reductive processes.

## 2 Materials and methods

### 2.1 Culture media, biochemicals and drugs

If not otherwise stated, all biochemical reagents were from Sigma (St Louis, MO, USA). Nitazoxanide (NTZ) was synthesized at the Department of Chemistry and Biochemistry, University of Bern, Switzerland (Ch. Leumann). 6-(7-nitro-2,1,3-benzoxadiazol-4-ylthio)hexanol (NBDHEX) was synthesized at the Department of Sciences and Chemical Technologies, University of Rome and kindly provided by M. Lalle (Department of Infectious, Parasitic and Immune-mediated Diseases, Rome, Italy). Albendazole (ALB), NTZ, metronidazole (MET), and NBDHEX were kept as 100 mM stock solutions in DMSO at −20 °C.

### 2.2 Axenic culture of *G. lamblia* trophozoites

Trophozoites from *G. lamblia* WB clone C6 wild-type and of the NTZ/MET resistant clone C4 were grown under anaerobic conditions in 10 ml culture tubes (Nunc, Roskilde, Denmark) containing modified TYI-S-33 medium as previously described (Clark and Diamond, 2002). C4 was routinely cultured in the presence of 50 μM NTZ. Subcultures were performed by inoculating 20 μl (wild-type) or 100 μl (C4) of cells from a confluent culture detached by cooling (Müller et al., 2006) to a new tube containing the appropriate medium.

### 2.3 Harvest and storage of *G. lamblia* trophozoites

For all experiments comparing wild-type to C4 trophozoites, the medium from confluent cultures was removed one day before the harvest and replaced with fresh medium without NTZ.

Trophozoites were detached by incubation on ice for 15 min followed by centrifugation (300 × g, 10 min, 4 °C). Pellets were washed twice with PBS and either stored at −20 °C (for functional assays) or used directly (determination of dinucleotides and ADP/ATP-ratio, whole-cell assays and RT-PCR).

### 2.4 Growth curves, determination of minimal inhibitory concentrations, stability of resistance

For all growth studies, *G. lamblia* WBC6 wild-type (WT) and the MET- and NTZ-resistant strain C4 were inoculated into culture tubes (10^4^ trophozoites per tube). To determine the respective growth curves, WT and C4 trophozoites were grown with 50 μM NTZ or with equal amounts of DMSO as a solvent control. At various time points, adhering cells were counted in a Neubauer chamber (Müller et al., 2006). To determine minimal inhibitory concentrations (MIC), WT and C4 trophozoites were inoculated in the presence of increasing amounts (dilution series by a factor 2) of the nitro compounds MET, NTZ or NBDHEX, and of ALB as a control. The tubes were incubated at 37 °C for 4 days. The MIC was determined by observing the wells under the microscope starting from higher to lower concentrations. The concentration at which the first living trophozoites were visible is given as the MIC.

### 2.5 Scanning and transmission electron microscopy

For scanning (SEM) or transmission (TEM) electron microscopy, trophozoites were harvested as described above and processed as described earlier (Müller et al., 2006), with the sole exception that UranyLess EM Stain (Electron Microscopy Sciences, Hatfield, PA) was used instead of uranyl acetate.

### 2.6 RNA analysis and quantification of expression by real-time PCR

For quantification of expression of characterized proteins by real-time PCR after reverse transcription (RT-PCR), trophozoites were grown and harvested as described above. RNA was extracted using the QIAgen RNeasy kit digestion (QIAgen, Hilden, Germany) according to the instructions by the manufacturer. RNA was eluted with RNase-free water and stored at −80 °C. First-strand cDNA was synthesized using the QIAgen OmniscriptRT kit (QIAgen, Hilden, Germany). After quantitative RT-PCR, expression levels were given as relative values in arbitrary units relative to the amount of actin. Quantitative RT-PCR was performed as described (Müller et al., 2008) using the primers listed in Table 1.

**Table 1:** Primers used in this study. Gene annotations and accession numbers were retrieved from GiardiaDB (giardiadb.org). The genes marked with an asterisk are lateral transfer candidates.

Name	Sequence	GiadiiaDB accession number
ACT_F	ACATATGAGCTGCCAGATGG	Actin related protein (GL50803_40817)
ACT_R	TCGGGGAGGCCTGCAAAC	
FDP_F	TGGGTGGAGCAACAGGGC	A-type flavoprotein; flavodiiron* protein (GL50803_10358)
FDP_R	TTACTGCTTAGGGGCGTTCT	
FlaHB_F	GGACAGAGAGGGCGAGGA	Flavohemoprotein* (GL50803_15009)
FlaHB_R	CTAATGGGAGGCCTTGAAG	
NO_F	GCACGACACGCATCATCC	NADH oxidase
NO_R	TTACAGTTTCATCAGCGTGG	(GL50803_9719)
NOLT_F	ACACGGACAGGCCTGGGT	NADH oxidase lateral transfer
NOLT_R	TCAGTCCTTCTTGTTTATCGCAC	candidate* (GL50803_33769)
GlNR1_F	CCTGCTGACAAGGCCGCA	Nitroreductase Fd-NR2* (GL50803_22677)
GlNR1_R	AACACCAATTACTTAAATGTAATG	
GlNR2_F	CTGCAGCTTCACTCAGAGA	Nitroreductase Fd-NR1* (GL50803_6175)
GlNR2_R	TTATTCCACAAACGTTACGTC	
NRfam_F	GGGAATACAAAATGACGGGG	Nitroreductase family protein* (GL50803_15307)
NRfam_R	GTACTCTTCTGTTTGGCGAG	
POR1_F	ATCCAACGCGACCCAGAAG	Pyruvate-flavodoxin-oxido-reductase (GL50803_17063)
POR1_R	GTTCACTGCTTACTCCGCC	
POR2_F	CTCGCACATGGTCCAGGG	Pyruvate-flavodoxin-oxido-reductase (GL50803_114609)
POR2_R	AGAGCCGCAGCCATCTCC	
TrxR_F	CGTTGGCCACGATCCCC	Thioredoxinreductase (GL50803_9827)
TrxR_R	TACTCCTGCATGGCAAGCC	

### 2.7 Whole-cell-assays

Oxygen consumption (OCR) and extracellular acidification rates (ECAR) were simultaneously determined (Divakaruni et al., 2014) using a Seahorse XFp device (Agilent, Santa Clara, CA). For each assay, WT or C4 trophozoites were harvested as described and suspended in PBS (2 × 10^7^ cells/ml), and the suspension was added to XFp cell culture miniplates (50 μl per well) containing 150 μl of a sterile NaCl 0.9% (w/v) solution. Plates were centrifuged (400 × g, 2 min, 20 °C) in order to ensure adhesion of the trophozoites. Then, the measurements were performed according to the instructions provided by the manufacturer. During the internal calibration of the XFp extracellular flux cartridge (ca. 20 min), the miniplates containing the trophozoites were incubated at 37 °C and then transferred into the device. OCR and ECAR rates were determined by averaging the rates obtained between 6 and 30 min after the start of the analysis and normalized to the protein contents of the cells.

To determine initial resazurin reduction rates, WT or C4 trophozoites were suspended in PBS or PBS containing 0.2% (w/v) glucose (10^5^ trophozoites per ml). 0.1 ml of this suspension were added to 96-well-plates. The assay was started by adding 0.1 ml of resazurin (20 mg/l) in PBS and the reduction of resazurin was quantified at 37 °C by fluorimetry with excitation at 530 nm and emission at 590 nm using a 96-well-multimode plate reader (Enspire; Perkin-Elmer, Waltham, MA).

### 2.8 Functional assays

Extracts were prepared from frozen pellets suspended in assay buffer containing 0.5% (v/v) Triton-X-100 and 1 mM phenyl-methyl-sulfonyl-fluoride. Nitroreductase activity was determined by measuring the formation of 7-amino-coumarin (Müller et al., 2015). The assay buffer (Tris/Cl^−^ 50 mM, pH 7) contained 7-nitrocoumarin (0.1 mM) as a substrate and NADH or NADPH (0.5 mM) as electron donors. The reaction was started by addition of the electron donor.

Pyruvate oxidoreductase assays were performed in potassium phosphate (100 mM, pH 7) containing sodium pyruvate (10 mM), coenzyme A (0.2 mM), MgCl_2_ (1 mM), and thiaminpyrophosphate (5 μM) as described (Hoffman et al., 2007) with the sole exception that thiazolyl blue tetrazolium chloride was used as final electron acceptor instead of benzyl viologen.

Ornithine-carbamyl-transferase was assayed in the direction of citrulline formation and citrulline was quantified as described (Schofield et al., 1992). This assay was slightly modified for the determination of citrulline by adding convenient amounts of cell-free extracts directly to the stop and colour development solution.

### 2.9 Determination of cofactor pool sizes and ratios

NAD(H) and NADP(H) contents were determined using commercial kits (NAD^+^/NADH-Glo™ and NADP^+^/NADPH-Glo™) according to the instructions provided by the manufacturer (Promega, Madison, WI). FAD was determined using a commercial kit (FAD colorimetric/fluorometric assay kit) according to the instructions provided by the manufacturer (Biovision, Milpitas, CA). The ADP/ATP-ratio was determined using a commercial kit (Biovision ADP/ATP Bioluminescence assay kit) according to the instructions provided by the manufacturer. For all assays, trophozoites were harvested as described, counted and freshly processed using the respective extraction buffers provided in the kits. The extraction buffers of all kits contained detergents and ensured an instaneous and >95% lysis of the trophozoites. The assays were run in quadruplicates in 96-well-plates containing the equivalent of 10^4^ cells per well. The mean values and standard errors of three independent assays normalized to the protein contents of the cells are shown.

### 2.10 Protein contents

Protein contents of cell-free extracts were determined by the Bradford method (Bradford, 1976) using a commercial kit (Biorad Laboratories, München, Germany). For the normalization of whole-cell-assays, the trophozoites were lysed in PBS containing 0.05% (v/v) Triton-X-100.

### 2.11 Statistics

Student's t-tests were performed using the software package R (R Core Team, 2012). Differences of the mean values with p < 0.01 were regarded as statistically significant.

## 3 Results

### 3.1 Growth and maintenance of resistance

**Fig. 1:**
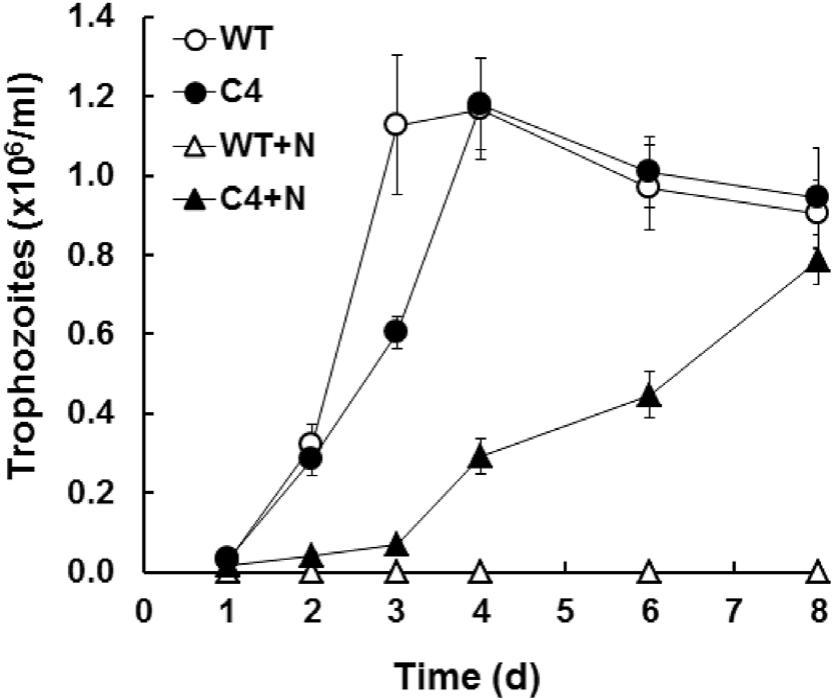
Growth of the nitro drug-resistant *G. lamblia* strain C4. At day 0, 10^4^ WBC6 (WT; white symbols) or C4 (black symbols) trophozoites were inoculated to normal culture medium (circles) or to medium containing 50 μM NTZ (+N; triangles). Mean values (+SE) from quadruplicate determinations are given.

In order to illustrate the resistance of the *G. lamblia* strain C4 derived from the wild-type WBC6, we determined the minimal inhibitory concentrations of three nitro compounds, namely MET, NTZ and NBDHEX on both strains. Whereas all three compounds inhibited the wild-type clone at MICs in the 10-μM-range, none of the compounds inhibited strain C4, and were ineffective even at 100 μM, the highest concentration used in this test. Conversely, the MIC for the benzimidazole ALB – thus a non-nitro drug – was similar in both strains (Table 2). In the absence of drugs, C4 trophozoites proliferated almost as rapidly as the WT trophozoites, reaching confluence after 4 d post inoculation. In the presence of 50 μM NTZ, thus in the medium used to maintain strain C4, the proliferation of resistant trophozoites was slower, and confluence was reached after approximately one week post inoculation (Fig. 1). Upon subsequent passages on drug-free medium, the resistance slowly declined, but was nevertheless maintained, as already published (Müller et al., 2008).

**Table 2:** Determination of minimal inhibitory concentrations (MICs) of serial dilutions of the nitro compounds MET, NTZ and NBDHEX and of ALB on WBC6 wild-type (WT) and C4 trophozoites. MICs were determined as described in Materials & Methods and are given in μM.

Compound	WBC6	C4
MET	12.5	>100
NTZ	3.1	>100
NBDHEX	12.5	>100
ALB	0.2	0.2

### 3.2 Ultrastructure

**Fig. 2:**
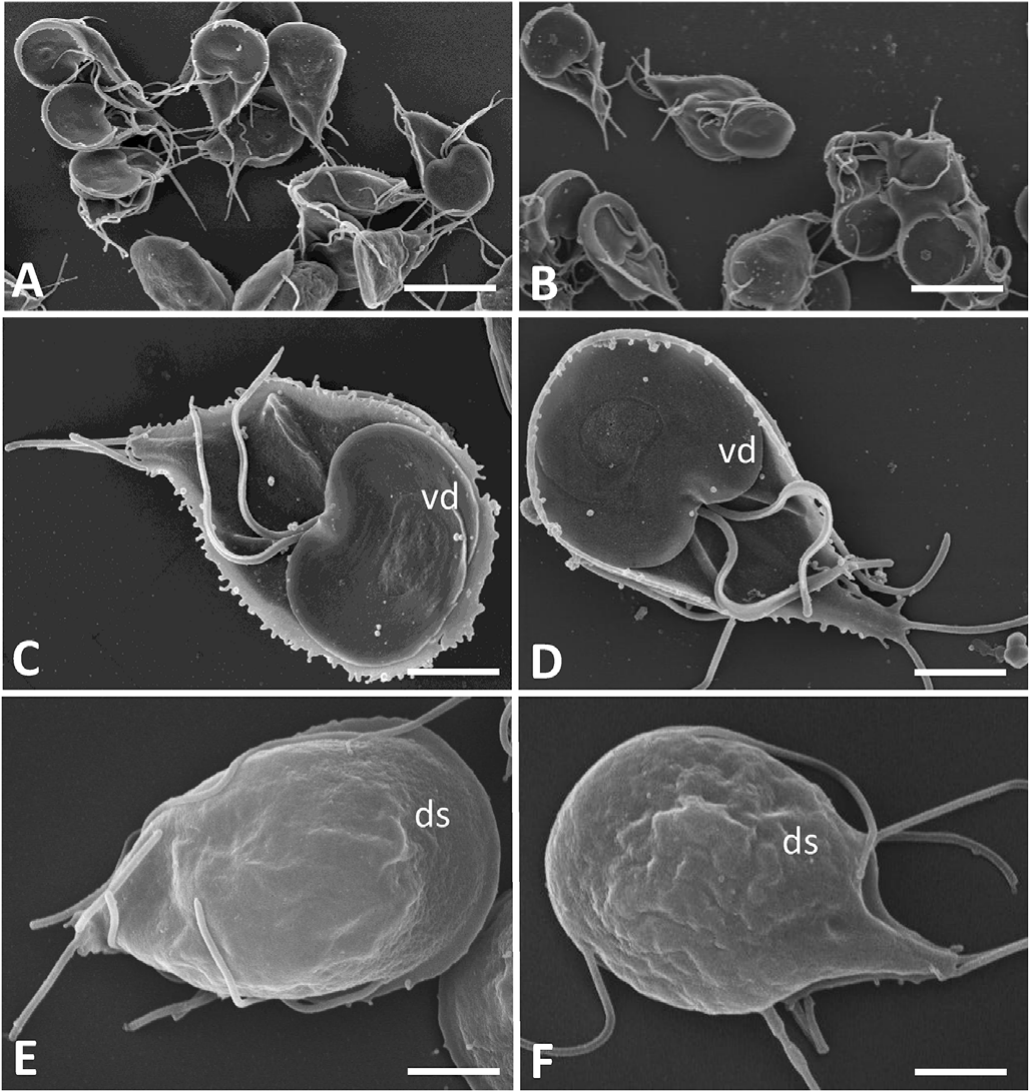
SEM of trophozoites of the *G. lamblia* strain C4 (A, C, E) and corresponding wild-type strain WBC6 (B, D E). Low magnification views in A and B, ventral side in C and D, dorsal side in E and F. ds = dorsal surface, vd = ventral disc. Bars in A and B = 9.3 μm; bars in C - F = 2.8 μm.

**Fig. 3:**
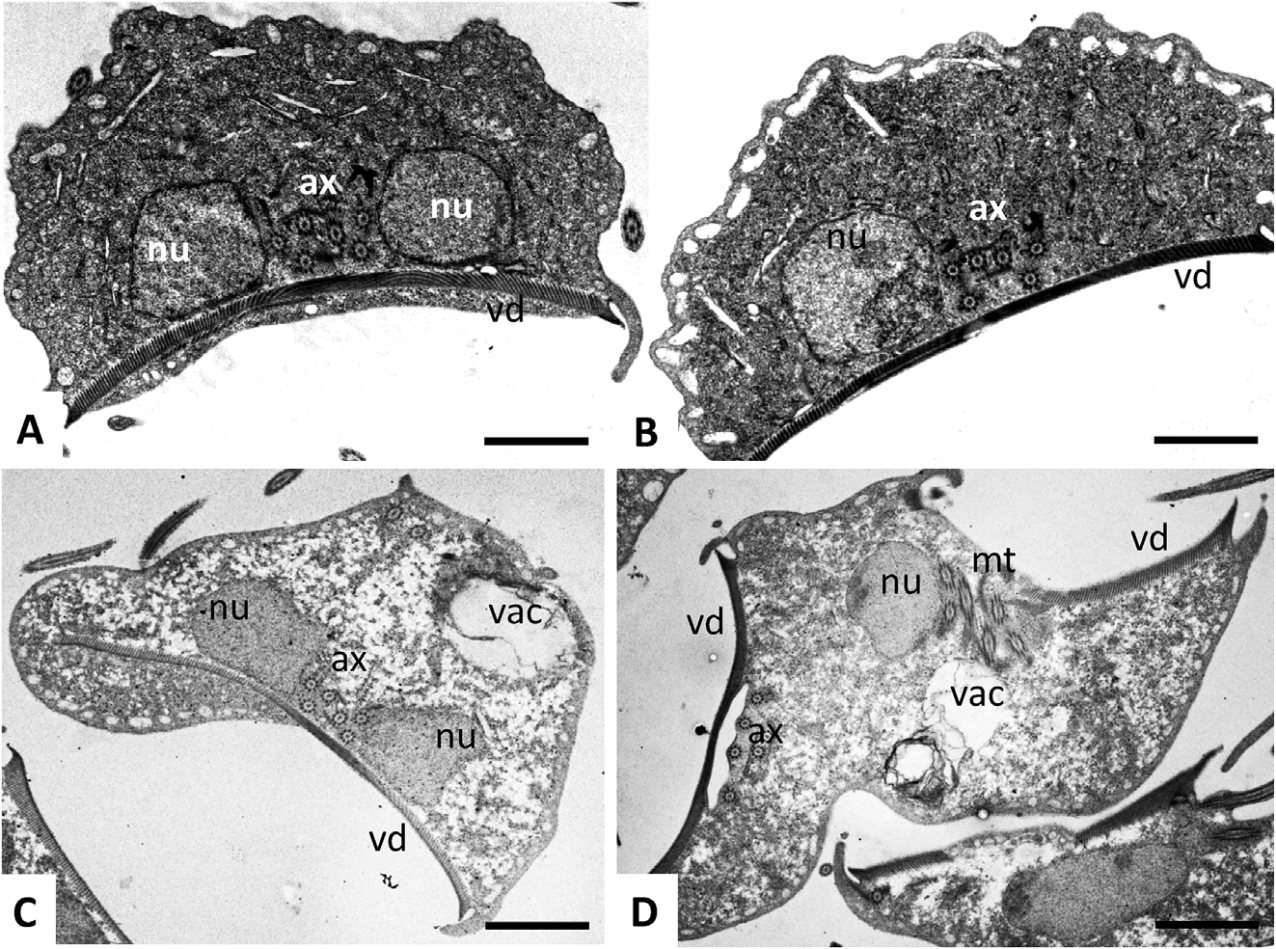
TEM of trophozoites of the *G. lamblia* wild-type WBC6 (A) and resistant strain C4 (B–D); ax = axonemes, mt = microtubules, nu = nucleus, vac = cytoplasmic vacuole, vd = ventral disc. Note in D a dividing trophozoite with two ventral discs. Bars in A and B = 2.8 μm; bars in C and D = 3.2 μm.

Both wild-type and strain C4 were fixed and processed for SEM, and inspection of specimens did not reveal morphological differences, neither on the ventral disc nor on the dorsal surface of the trophozoites (Fig. 2), nor did the sizes of the trophozoites differ. The sizes, as measured from SEM micrographs as shown in Fig. 2 were for WT trophozoites 13.2 ± 0.5 μm for the long axis and 7.9 ± 0.2 μm for the small axis. The values of the same parameters of C4 trophozoites were 12.9 ± 0.4 μm and 8.6 ± 0.5 μm, respectively (n = 5). TEM did also not indicate dramatic differences between the two stains. All characteristic features of trophozoites including the ventral disc and the axonemes located between the two nuclei appeared structurally unaltered (Fig. 3). However, in approximately 10% of the trophozoites of strain C4, cytoplasmic vacuolization could be observed, often in combination with a less electron-dense cytoplasm. This feature was virtually absent in WT trophozoites.

### 3.3 Oxygen consumption and resazurin reduction rates

**Fig. 4:**
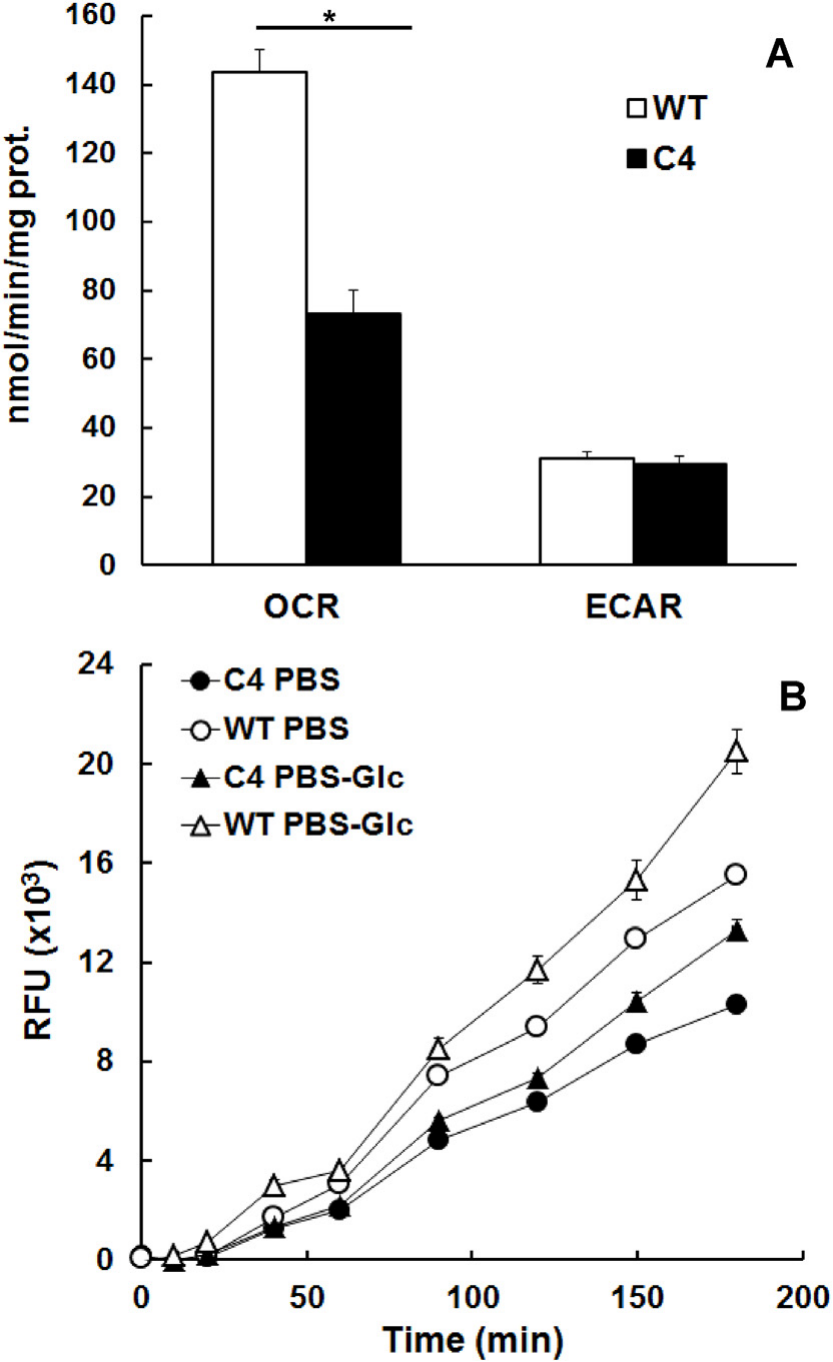
Whole cell assays with WBC6 (WT; white symbols) and nitro drug resistant (C4; black symbols) trophozoites. A, oxygen consumption (OCR) and extracellular acidification (ECAR) rates determined in a Seahorse XFp device. Mean values (±) SE for three independent assays are given (*, p < 0.01). B, resazurin reduction. Mean values (+SE) from quintuplicate determinations in 96-well-plates (10^4^ trophozoites per well) are given. For both strains, the rates were determined in absence (circles) and presence (triangles) of glucose. Both assays were performed as described in Materials and Methods.

In order to see whether the results obtained on nitro reduction can be extended to metabolic processes involving other electron acceptors, we investigated oxygen consumption and resazurin reduction, both methods using intact cells. Oxygen consumption rates were significantly lower in C4 trophozoites reaching ca. 50% of the wild-type levels. Conversely, extracellular acidification rates were similar in both strains (Fig. 4 A, see Fig. S1 for a typical experiment). Similar observations could be made by offering resazurin as an electron acceptor. C4 trophozoites had lower resazurin reduction rates (61 RFU/min) than WT trophozoites (92 RFU/min). For both strains, the rates were increased in the presence of glucose (76 vs. 114 RFU/min; Fig. 4 B).

### 3.4 Messenger RNA levels of enzymes involved in nitro reduction

In a next step, we investigated the mRNA levels of a panel of selected genes, including the gene coding for nitroreductase GlNR1. No differences in transcription levels could be detected between strain C4 and WT trophozoites, with the exception of GlNR1 mRNA, whose levels were significantly lower in C4 trophozoites than in WT trophozoites, thus confirming previous results (Müller et al., 2008, 2013; Nillius et al., 2011). The mRNA levels of other genes involved in nitro reduction including both POR isoforms were the same in both strains (Table 3).

**Table 3:** Expression of *G. lamblia* enzymes involved in reduction of nitro compounds in the MET/NTZ resistant strain C4 as compared to the wild-type (WT) WBC6. Trophozoites were grown to confluence and harvested as described. To determine mRNA levels, RNA was extracted and reverse transcribed into cDNA. Transcripts were quantified in relation to actin. Mean values ± SE are given for four independent samples. Values marked by asterisks are significantly different to the control (paired *t*-test, two-sided; *, P < 0.001). The full names of the genes, the accession numbers and the primers are given in Table 1 n.d., not detected.

Gene	mRNA levels	
	WT	C4
FDP	1.9 ± 0.0	1.8 ± 0.1
FlaHb	1.1 ± 0.01	0.1 ± 0.01
NR1	0.18 ± 0.03	0.03 ± 0.01*
NR2	0.13 ± 0.01	0.16 ± 0.02
NRfam	0.05 ± 0.01	0.07 ± 0.003
POR1	1.5 ± 0.2	2.1 ± 0.2
POR2	3.1 ± 0.4	5.7 ± 0.01
TrxR	4.4 ± 0.01	4.6 ± 0.3
NO	8.4 ± 0.5	8.3 ± 0.5
NOLT	8.8 ± 0.7	14.7 ± 0.6

### 3.5 Nitroreductase activity and pool sizes of cofactors of nitro-reducing enzymes

**Fig. 5:**
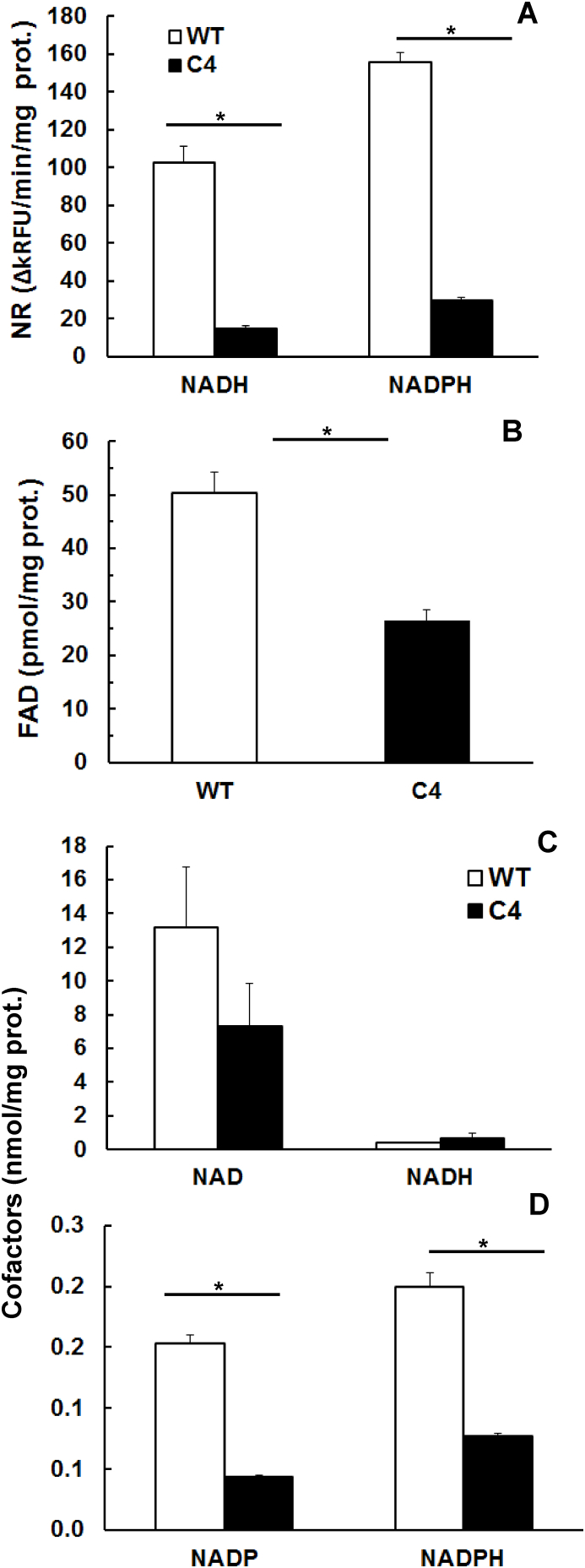
Nitroreductase (NR) activities and cofactor contents in WBC6 (WT; white bars) and nitro drug-resistant (C4; black bars) trophozoites. A, NR activity determined with NADH or NADPH as cofactors. B; FAD contents; C, NAD(H) contents; D, NADP(H) contents. All assays were performed as described in Materials and Methods. Mean values (±) SE for three independent assays are given (*, p < 0.01).

According to the current knowledge on the mode of action of nitro drugs, reduction of nitro groups to more toxic intermediates should be impaired in resistant strains as compared to wildtype strains. To verify this hypothesis, we measured 7-nitrocoumarin reductase activity in total cell extracts of WT and C4 trophozoites using either NADH or NADPH as electron donors. As controls, we determined pyruvate-oxidoreductase (POR) activity and – as a not-oxidoreductase control - ornithine carbamyl transferase (OCT). POR activity did not significantly differ in extracts from both lines (Table 4). In contrast, nitroreductase activity was markedly reduced in extracts of C4 trophozoites, reaching only ca. 20% of the activity level in WT extracts, regardless which electron donor had been offered (Fig. 5 A). Interestingly, the second enzyme activity that we included as a control, namely OCT, was significantly lower in C4 extracts compared to extracts of WT trophozoites (Table 4). This observation prompted us to investigate the levels of citrulline, the product or educt of OCT. C4 trophozoites, contained less citrulline, namely 2.7 ± 0.3 nmol/mg protein, compared to 8.3 ± 1.0 nmol/mg protein in WT trophozoites.

**Table 4:** Specific enzyme activities in crude extracts of trophozoites of the MET/NTZ resistant strain C4 and of the wild-type (WT) WBC6. Pyruvate-oxido-reductase and ornithine-carbamyl-transferase activities were determined as described in Materials and Methods. Mean values ± SE are given for three independent extracts. Values marked by asterisks are significantly different to the control (paired *t*-test, two-sided; *, p < 0.01).

Activity	Unit	WT	C4
Pyruvate-oxido-reductase	ΔmA590/min/mg protein	34.3 ± 6.0	46.6 ± 6.6
Ornithine-carbamyl-transferase	nkat/mg protein	94.5 ± 0.7	37.1 ± 6.5*

Since – except for GlNR1 – the expression levels for other known or alleged nitro-reducing enzymes were similar in both strains (cf. Table 2), it was of interest to determine whether the pool size of the prosthetic group responsible for electron transfer to nitro groups, to oxygen and to xenobiotics with a similar redox potential, FAD, was altered in the resistant strain. In-terestingly, the FAD level in C4 trophozoites amounted to only about 50% of the level found in WT trophozoites (Fig. 5 B).

In a next step, we investigated the pool sizes and ratios of the nicotinamide-dinucleotide co-factors involved in electron transfer, and of the ADP/ATP-ratio as a marker for the energy status. NAD(H) was by far more abundant in trophozoites than NADP(H) and exhibited a higher degree of variation between independent preparations (Fig. 5C and D). The levels of NAD and NADH did not significantly differ between WT and C4 trophozoites (Fig. 5 C), the NADP and NADPH levels were, however, significantly reduced in C4 trophozoites (Fig. 5 D).

The ratios of NAD versus NADH in trophozoites of both strains balanced strongly in favour of NAD, and were slightly, but not significantly, higher in WT than in C4 trophozoites (p < 0.1). In contrast, the NADPH/NADP ratios were, however, closer to one and significantly increased in C4 trophozoites. In WT trophozoites, the ADP/ATP-ratio was close to one and significantly increased in C4 trophozoites (Table 5). The absolute ATP contents were 24.1 ± 3.1 nmol/mg protein in WT versus 21.8 ± 1.9 nmol/mg protein in C4 trophozoites. The absolute ADP contents were 25.0 ± 5.7 nmol/mg protein in WT vs 29.8 ± 4.2 nmol/mg protein in C4 trophozoites. The differences were not significantly different.

**Table 5:** Ratios of nicotinamide dinucleotides and of ADP/ATP. All assays were performed as described in Materials and Methods. The assays were run in quadruplicates in 96-well-plates containing the equivalent of 10^4^ cells per well. The mean values and standard errors of three independent assays normalized by the protein contents of the cells are shown. Values marked by asterisks are significantly different to the control (paired *t*-test, two-sided; *, p < 0.01).

Ratio	WT	C4
ADP/ATP	1.04 ± 0.03	1.4 ± 0.1*
NAD/NADH	32.3 ± 6.3	12.6 ± 2.0
NADPH/NADP	1.31 ± 0.03	1.77 ± 0.02*

## 4 Discussion

In the present study, we have investigated physiological aspects of resistance formation in *G. lamblia* using the nitro drug-resistant strain C4 and its isogenic wild-type WBC6 as a “model system”. Trophozoites of the two strains did not differ markedly with respect to cell shape and ultrastructural characteristics, thus physiological parameters such as enzyme activities and metabolite content could be compared.

C4 trophozoites exhibit similar mRNA expression levels of genes coding for enzymes invoved in nitro and/or O_2_-reduction, including GlNR2 (Müller et al., 2013) and a homologous protein without N-terminal ferredoxin domain (NRfam), other flavoproteins like flavodiiron protein and flavohemoglobin (Di Matteo et al., 2008; Vicente et al., 2009; Mastronicola et al., 2010; Rafferty et al., 2010), thioredoxin reductase (Brown et al., 1996b; Leitsch et al., 2016; Camerini et al., 2017), NADH oxidase (Brown et al., 1996a; Li and Wang, 2006; Castillo-Villanueva et al., 2016) or the two POR isoforms (Leitsch et al., 2011). The only enzyme shown to exhibit significantly decreased mRNA levels in C4 trophozoites is GlNR1. This result is in good agreement with data obtained with three other MET-resistant strains (Ansell et al., 2017). The significant decrease of nitroreductase activity in cell-free extracts of C4 trophozoites, thus has other origins, namely either post-transcriptional downregulation or lack of essential cofactors. Since the electron donors NAD(P)H are provided in excess in the functional assay, and since the most relevant nitro-reducing enzymes are flavoproteins, the incorporation of the prosthetic group FAD may be critical. As previously suggested (Leitsch et al., 2011; Ansell et al., 2015), the reduction of FAD levels may thus constitute an important physiological mechanism to avoid the formation of toxic nitro intermediates and/or radicals. Since NADH oxidases also contain FAD (Brown et al., 1996a), it is not surprising that the oxygen consumption of C4 trophozoites is reduced, as well. This differs from results of a former study where no differences in oxygen consumption between MET-sensitive or - resistant clinical isolates could be observed (Ellis et al., 1993). It is unclear, however, to which extent these results can be extrapolated since the cells have been grown under different conditions. Furthermore, we have to consider the possibility that FMN and not FAD is the cofactor of some enzymes that may be involved in nitroreduction (Ansell et al., 2015).

The surprising observations concerning OCT activity and citrulline contents indicate, however, that besides redox processes also other metabolic mechanisms are affected in resistant strains. OCT plays a critical role in giardial energy metabolism (Schofield et al., 1990, 1992; Edwards et al., 1992) and is up-regulated on mRNA and protein levels in ALB-resistant trophozoites (Paz-Maldonado et al., 2013). The fact that C4 trophozoites have a lower OCT activity may be an indication for a diminution of energy production and of intermediate metabolism. Lower citrulline pool size (Knodler et al., 1994), higher ADP/ATP and NADPH/NADP^+^ ratios and lower growth rates indicate the same.

Taken together, the metabolic parameters that we have investigated support the thesis that resistance formation to nitro drugs in C4 is due to a reduction of nitro drug activation rather than to a detoxification of nitro radicals. Expression studies have revealed a downregulation of the nitroreductase NR1, a potential activator of nitro compounds (Nillius et al., 2011; Müller et al., 2015) whereas enzymes involved in nitro radical detoxification such as the nitroreductase NR2 (Müller et al., 2013, 2015), flavohemoglobin (Mastronicola et al., 2010), and flavodiiron protein (Vicente et al., 2009) are not affected. Conversely, other resistant strains show expression patterns suggesting that both mechanisms are involved in resistance formation (Ansell et al., 2017). Aerobic resistance, thus quenching of nitro radicals by O_2_ as observed in the microaerophilic *Trichomonas* sp. (Tachezy et al., 1993) can be excluded for *Giardia* growing under strictly anaerobic conditions.

To sum up, resistance formation exhibits striking similarities to metabolic adaptation processes to environmental distress, and, in this case, is less likely caused by mutations of single intracellular targets. This may be anchored in the evolutionary history of this protozoan parasite, which must face dietary shifts of its omnivorous hosts ranging from a carbohydrate-rich to a red meat-rich diet, resulting in an accumulation of nitrosamines and other reactive nitrogen species (Hughes et al., 2001; Kuhnle et al., 2007; Joosen et al., 2009). These compounds may be generated at biologic heme centers mediating e.g. the nitration of phenol and tryptophan (Casella et al., 2002; Lunn et al., 2007). When using nitro drugs, the treatment success would then be only guaranteed by an immediate increase from zero to a concentration above the MIC until complete parasite clearance. A step-wise increase of sublethal drug concentrations would result in adaptation as has been easily observed in the generation of the resistant lab strains by us and by other groups. The biochemical trigger of this adaptation is unknown. Since “resistance” formation has been shown to correlate with antigenic variation (Müller et al., 2008; Ansell et al., 2017) and since antigenic variation is due to epigenetic changes (Kulakova et al., 2006) at the post-transcriptional level (Prucca and Lujan, 2009), we may assume that the metabolic changes observed in this study may be – at least in part - the result of epigenetic changes, as well. This would explain the reversibility of “resistance” upon subcultures in the absence of drugs (Müller et al., 2008). Thus, nitro drug resistance shares some, but not all features (like e.g. elevated MICs) with the concept of “tolerance” as defined with respect to antibiotic treatment of bacteria (Brauner et al., 2016). As a consequence, we suggest replacing the term “resistance” by “tolerance” to nitro drugs as a special case of physiological plasticity towards environmental distress and suggest reserving the term “resistance” to genotypical changes such as point mutations of targets or acquisition of drug degrading enzymes by lateral transfer.

## Conflicts of interest

The authors declare that there is no conflict of interest.
