# Supplementary material for: Physiological aspects of nitro drug resistance in Giardia lamblia
Source: Int J Parasitol Drugs Drug Resist. 2018 Apr 28;8(2):271–7. doi: 10.1016/j.ijpddr.2018.04.008 (PMC6039359; doi:10.1016/j.ijpddr.2018.04.008)
Supplement: Supplementary material [file mmc2.docx]

**Supplemental data**


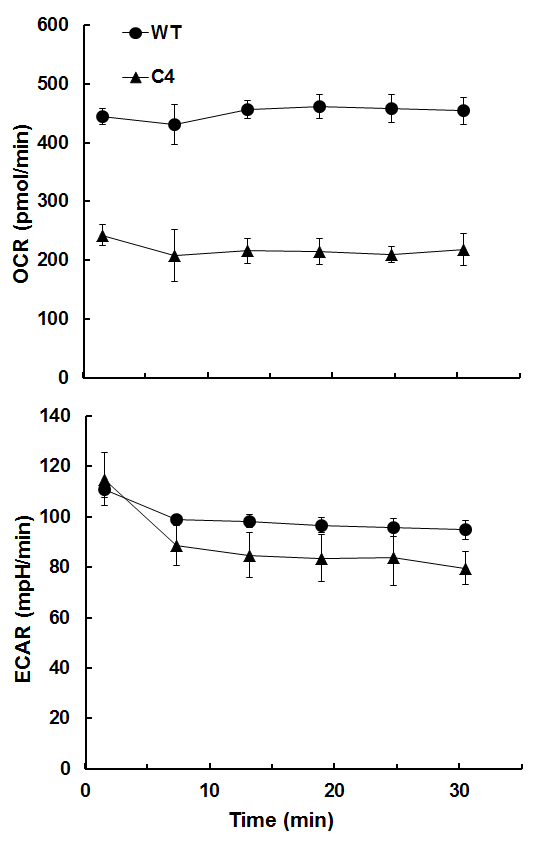


**Fig S1.** Representative data set of a series of oxygen consumption rate (OCR) and extracellular acidification rate (ECAR) measurements using the Seahorse XFp device. *G. lamblia* WBC6 (WT) and nitro-drug resistant (C4) trophozoites were processed as described in Materials & Methods. OCR and ECAR were determined in 6 measurement cycles each cycle comprising the following steps: i.) equilibration with ambient air by gentle mixing (2 min), ii.) pause (10 sec), iii.) measurement of the rates (3 min). Mean values and standard deviations of the rates as calculated after each step correspond to triplicates. Background rates obtained from wells containing buffer only (duplicates) were close to zero and have been subtracted at each step. As a result of one experiment, the rates from steps 2 to 6 have been averaged.
